# Acute Hypoglycemic and Antidiabetic Effect of Teuhetenone A Isolated from *Turnera diffusa*

**DOI:** 10.3390/molecules22040599

**Published:** 2017-04-08

**Authors:** Aída Parra-Naranjo, Cecilia Delgado-Montemayor, Alejandra Fraga-López, Gabriela Castañeda-Corral, Ricardo Salazar-Aranda, Juan José Acevedo-Fernández, Noemi Waksman

**Affiliations:** 1 Departmento de Química Analítica, Facultad de Medicina, Universidad Autónoma de Nuevo León, Monterrey, N.L., C.P. 64460, Mexico; ap._183@hotmail.com (A.P.-N.); ceciliadmontemayor@gmail.com (C.D.-M.); jany1524@hotmail.com (A.F.-L.); salazar121212@yahoo.com.mx (R.S.-A.); 2 Depto. de Fisiología y Fisiopatología, Facultad de Medicina, Universidad Autónoma del Estado de Morelos, Calle Leñeros s/n, Col. Los Volcanes, Cuernavaca Mor. C.P. 62350, Mexico; gabriela.castaneda@uaem.mx (G.C.-C.); juan.acevedo@uaem.mx (J.J.A.-F.)

**Keywords:** diabetes mellitus, damiana, teuhetenone A, antidiabetic, *Turnera diffusa*

## Abstract

Diabetes mellitus is a chronic degenerative disease that causes long-term complications and represents a serious public health problem. *Turnera diffusa* (damiana) is a shrub that grows throughout Mexico and is traditionally used for many illnesses including diabetes. Although a large number of plant metabolites are known, there are no reports indicating which of these are responsible for this activity, and this identification was the objective of the present work. Through bioassay-guided fractionation of a methanolic extract obtained from the aerial part of *T. diffusa*, teuhetenone A was isolated and identified as the main metabolite responsible for the plant’s hypoglycemic activity. Alpha-glucosidase inhibitory activity and cytotoxicity of this metabolite were determined. Hypoglycemic and antidiabetic activities were evaluated in a murine model of diabetes in vivo, by monitoring glucose levels for six hours and comparing them with levels after administering various controls. Teuhetenone A was not cytotoxic at the tested concentrations, and did not show inhibitory activity in the glucosidase test, and the in vivo assays showed a gradual reduction in glucose levels in normoglycemic and diabetic mice. Considering these results, we suggest that teuhetenone A has potential as an antidiabetic compound, which could be further submitted to preclinical assays.

## 1. Introduction

Type 2 diabetes mellitus (T2DM) is a chronic degenerative disease characterized by hyperglycemia, mainly due to defects of insulin secretion and/or action. The disorder is associated with an imbalance of the glycemic index and glucose intolerance, which increases the risk of individuals progressing to T2DM. Complications from diabetes, such as coronary artery and peripheral vascular disease, stroke, diabetic neuropathy, risk of ulcers and amputations, renal failure, sexual dysfunction, and blindness are resulting in increasing disability, reduced life expectancy, and enormous health costs for virtually every society [1,2].

T2DM is a leading cause of morbidity and mortality in industrialized and developing countries, resulting in part from high-carbohydrate and high-fat diets that lead to excess weight and/or obesity, as well as reduced physical activity. The disease is endemic in low- and middle-income countries. In 2015, around two-thirds of all individuals with diabetes lived in such countries. The consequences of high-carbohydrate and high-fat diets are most severe in communities in developing countries, where poverty represents an additional problem [3,4].

T2DM is a serious public health problem. According to the World Health Organization, more than 422 million people worldwide are affected [5]. Its prevalence is increasing rapidly, and it is estimated that the number affected will double by 2040. Diabetes is one of the major causes of morbidity and mortality worldwide. In 2015, five million people died from complications of diabetes, making the disease and its complications the leading cause of death in most countries. In Mexico, 11.5 million people have diabetes, and Mexico is among the 10 countries with the highest prevalence of this disease. According to the National Institute of Statistics and Geography data of 2015, the percentage of deaths attributed to diabetes in Mexico is 15%, placing it as one of the main causes of mortality in the country [6].

T2DM is a complex disease, and a key component of the management process is to reinstate glycemic control to as near normal levels as is practicable, safe, and comfortable for the patient. This goal is achieved by lifestyle adaptation, particularly in regards to diet and exercise, health education, and pharmacological treatment. The last approach includes insulin and hypoglycemic agents (including sulfonylureas, biguanides, thiazolidinediones, and α-glucosidase inhibitors), and focuses on increasing insulin levels, improving sensitivity to the hormone in tissues, or reducing the rate of carbohydrate absorption from the gastrointestinal tract [7,8]. However, despite being effective, these agents provide inadequate glycemic control, and/or are associated with side effects or non- adherence. The most frequently-reported adverse effects of these agents include hypoglycemia, weight gain, edema, and heart failure [9]. Alternatives are being sought for affected diabetic patients, for whom the development of new hypoglycemic therapies is desirable.

Plants have formed the basis of traditional medicine systems around the world for thousands of years. Even in modern systems, ethnobotanical treatments continue to play an important role in health care. About 80% of the population in developing countries rely on traditional medicine for their health care. In Mexico, there is a great diversity of medicinal plants. Andrade et al. reported on 306 species of Mexican plants used as hypoglycemic agents [10]. Despite the large number of plants that have been reported for their empirical use as antidiabetics, only a small percentage of these plants have been studied in a systematic way, and the molecules responsible for the biological activity of most of them are unknown.

Within the list of Mexican plants used as hypoglycemic agents, *Turnera diffusa* (damiana) attracted our attention. This shrub grows in arid and semiarid regions of South America, Mexico, the United States, and the West Indies. Throughout history, various properties have been attributed to this plant. Usually, the leaves of the plant are used to prepare a decoction [11,12]. Phytochemical investigations have been conducted to isolate and identify some components present in the plant, among which are flavonoids, sesquiterpenes, triterpenes, polyterpenes, fatty acids, and xanthine-derived sugars [13,14]. However, few reports exist [15,16,17,18] that provide evidence of its use for hypoglycemia. Therefore, the objective of the present study was to determine the basis of such use, by identifying the compounds responsible for its hypoglycemic activity.

## 2. Results

### 2.1. Isolation and Identification

**Figure 1:**
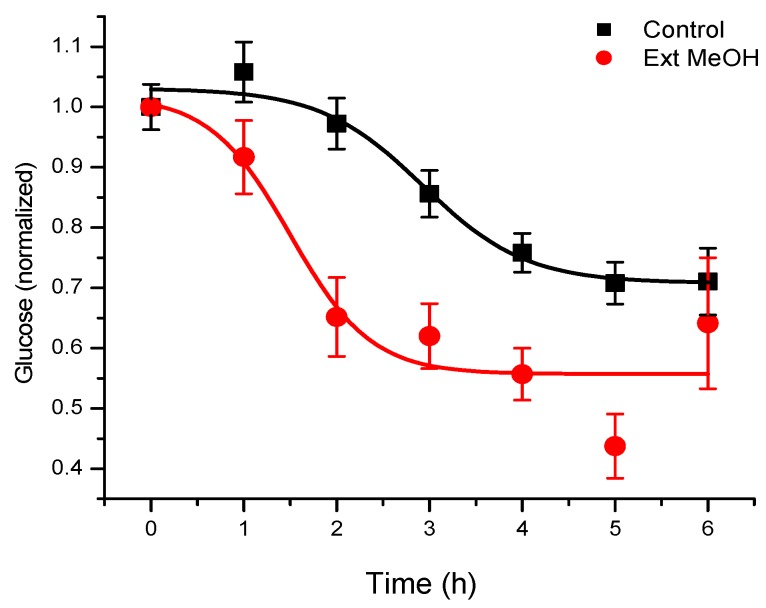
Hypoglycemic effect of damiana methanolic extract on adult CD1 mice. Dosage of 0.5 mg/kg administered intraperitoneally. *n* = 5, Mean ± standard error (SE).

**Figure 2:**
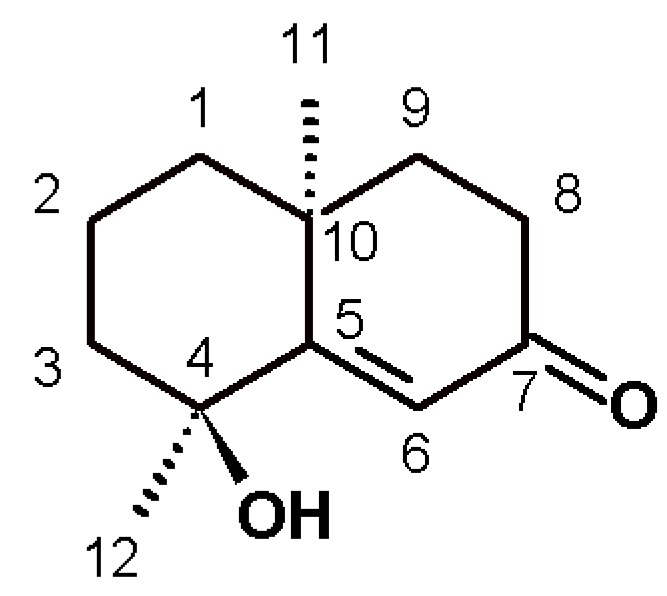
Structure of teuhetenone A.

In the first pharmacological approach to *T. diffusa* derivatives, the hypoglycemic activity of methanolic extracts of the plant was investigated in normoglycemic mice, as described in the Materials and Methods section (Figure 1). The results obtained corroborated the use of this plant for hypoglycemia. By means of a bioassay-guided fractionation, we determined that the terpenoid-rich fraction was responsible for the activity. In this fraction, we found a main active component (Figure 2), which was isolated and identified by spectroscopy, comparing spectral data with those reported in the literature.

Teuhetenone A was obtained as pale yellow oil (AcOEt), 95.37% chromatographic purity; [α]${}_{\text{D}}^{25}$ + 31 (*c* 0.1, MeOH), ^1^H-NMR(CDCl_3_, 400 MHz) δ_H_ 6.35 (1H, s, H-6), 2.57 (1H, ddd, J=17.9, 14.8, 5.2 Hz, H-8b), 2.38(1H, m, H-8a), 1.98 (1H, m, H-3b), 1.90 (1H, m, H-9b), 1.79 (1H, m, H-9a), 1.73 (2H, m, H-2), 1.66 (1H, m, H-1b), 1.58 (1H, m, H-3a), 1.43 (3H, s, H-12), 1.37 (1H, m, H-1a) y 1.31 (3H, s, H-11). ^13^C-NMR (CDCl_3_, 400MHz) δ_C_ 200.7 (C, C-7), 175.3 (C, C-5), 122.7 (CH, C6), 72.6 (C, C-4), 42.4 (CH_2_, C-3), 41.0 (CH_2_, C-8), 40.9 (CH_2_, C-1), 36.4 (C, C-10), 34.1 (CH_2_, C-9), 29.9 (CH_3_, C12), 24.7 (CH_3_, C-11), 19.7 (CH_2_, C-2). ESI/MS ^m^/_z_ 195 [M+H]^+^.

### 2.2. Alpha-Glucosidase Inhibitory Activity of Teuhetenone A

The isolated teuhetenone A was analyzed to determine its inhibitory activity on α-glucosidase. The results of this experiment are shown in Table 1.

### 2.3. Cytotoxic Activity of Teuhetenone A

The cytotoxicity of teuhetenone A was evaluated on a Vero cell line. No concentration tested was toxic to cells, so its CC_50_ was considered greater than 500 μg/mL.

### 2.4. Hypoglycemic Activity of Teuhetenone A in CD1 Mice

**Figure 3:**
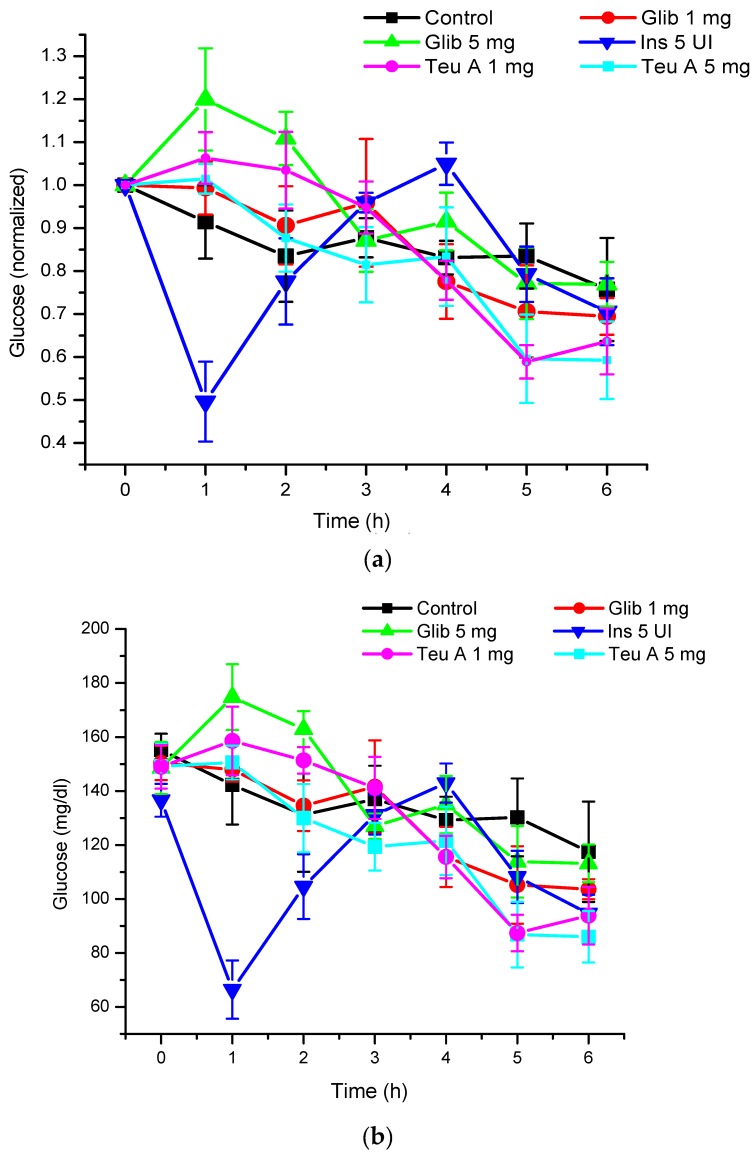
Hypoglycemic effect of insulin, glibenclamide, and teuhetenone A in adult CD1 mice. Glib - glibenclamide; Teu A - teuhetenone A; Ins - insulin. *n* = 5, Mean: ±SE. (**a**) Graph with normalized values; (**b**) Graph with unnormalized values. Standardized blood glucose for baseline glycemia to compare the kinetics of each experimental group.

Under the experimental conditions used, levels of glycemia were decreased by about 20% for six hours of fasting in CD1 mice treated with a vehicle control, and 40% after administration of teuhetenone A intraperitoneally, with no difference in the doses evaluated (1 and 5 mg/kg). Under the same conditions, insulin at 5 IU/kg had a hypoglycemic activity of 50% (Figure 3).

**Figure 4:**
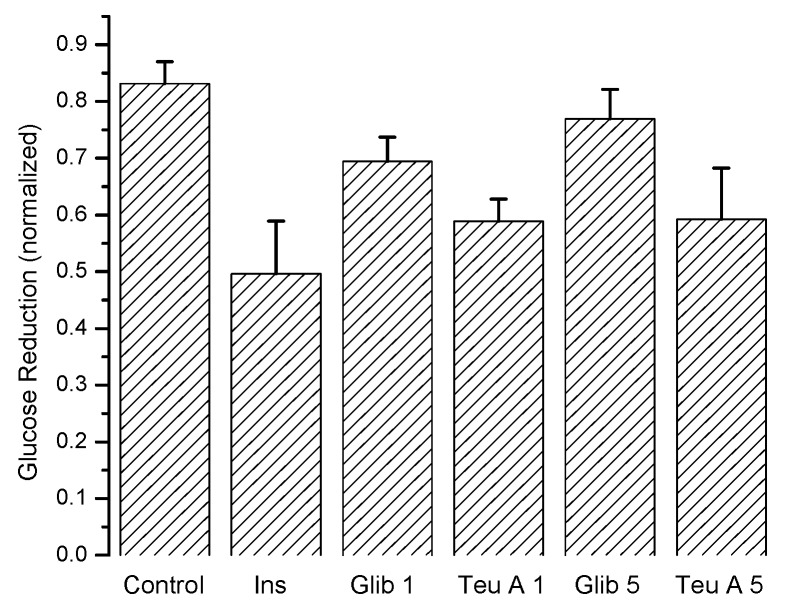
Maximum hypoglycemic effect of insulin, glibenclamide, and teuhetenone A in adult CD1 mice. Vehicle: water; Ins – insulin; Glib 1 - glibenclamide (1 mg/kg); Glib 5 - glibenclamide (5 mg/kg); Teu A 1 - teuhetenone A (1 mg/kg); Teu A 5 - teuhetenone A (5 mg/kg). *n* = 5, Mean ± SE.

The kinetics obtained with teuhetenone A were similar to those observed after intraperitoneal administration of the insulin secretagogue glibenclamide (1 and 5 mg/kg); however, the hypoglycemic effect was higher than that with glibenclamide, a commercial hypoglycemic agent (Figure 4).

### 2.5. Antidiabetic Activity of Teuhetenone A

**Figure 5:**
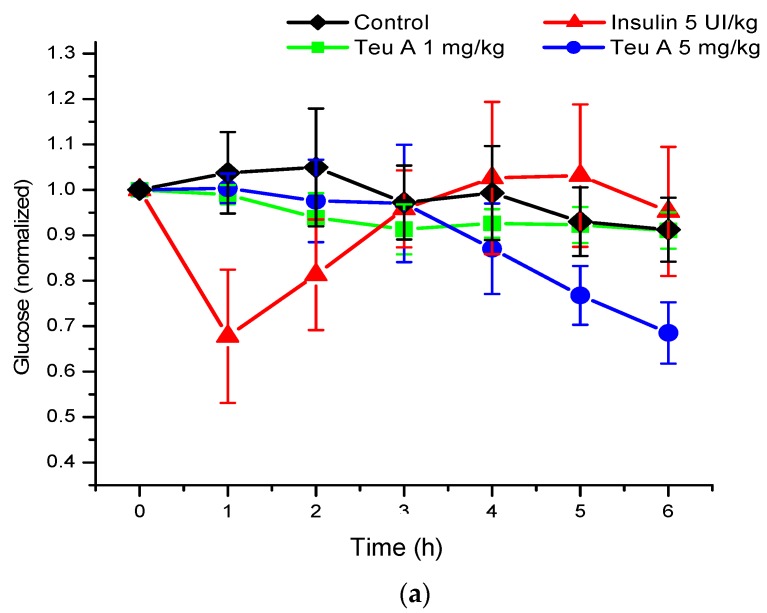

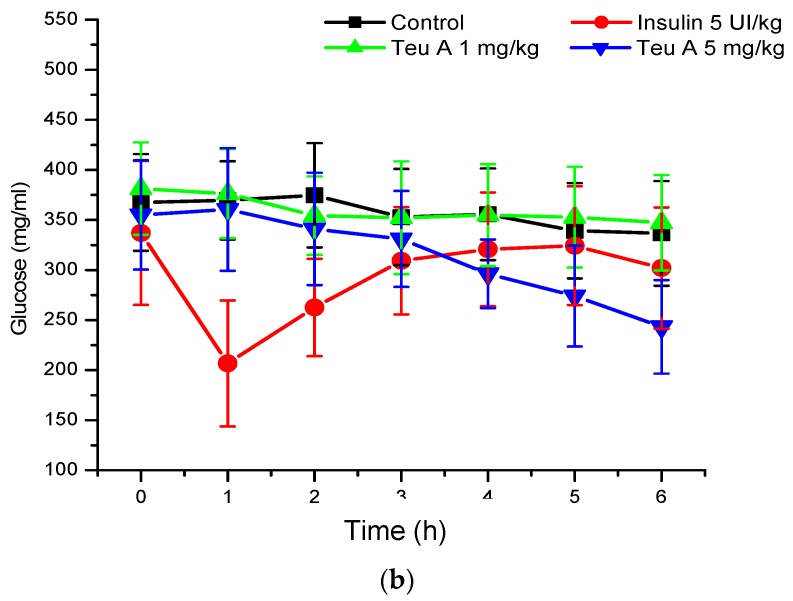
Antidiabetic effect of insulin and teuhetenone A in diabetic mice induced with 200 mg/kg of alloxan. *n* = 5, Mean ±SE. (**a**) Graph with normalized values; (**b**) Graph with unnormalized values. Standardized blood glucose for baseline glycemia to compare the kinetics of each experimental group.

In the murine model of diabetes used in the present study, insulin secretion was limited by the pancreotoxic effect of alloxan, so glycemia increased from 140–155 to 340–380 mg/dL (Figure 5), and insulin administration (5 IU/kg) reduced blood glucose by 35% at the time of administration. As observed in normoglycemic mice, glucose kinetics observed after insulin administration showed that the effect was maximal at the time of administration, and that glucose levels reverted during the evaluation. However, in the mouse model of diabetes, the vehicle control barely reduces glucose levels by 10%. By contrast, with the effect observed in normoglycemic mice, the effect of teuhetenone A was dose-dependent in diabetic mice, having no effect at 1 mg/kg but being active at 5 mg/kg. At the higher dose, the antidiabetic effect was similar to that observed with insulin, and glucose levels did not reverse during the period evaluated, being maximal at six hours after teuhetenone A administration (Figure 3 and Figure 5).

**Figure 6:**
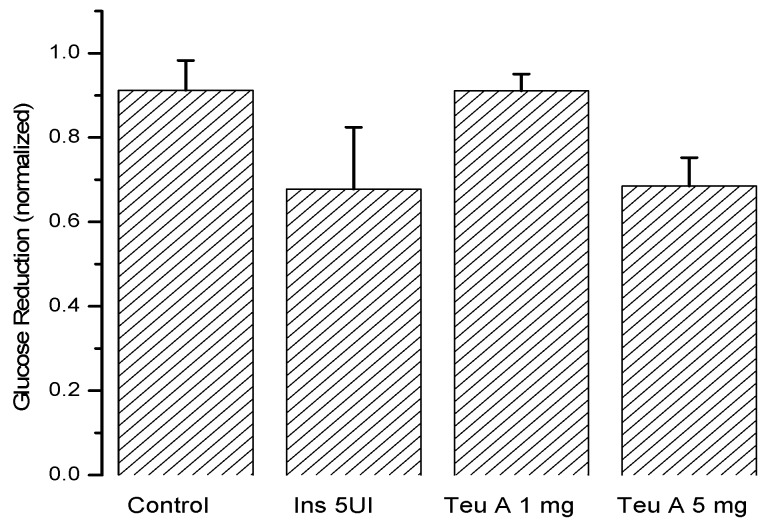
Maximum antidiabetic effect of insulin and teuhetenone A in diabetic mice induced with 200 mg/kg of alloxan. *n* = 5, Mean ± SE.

Figure 6 shows the maximum antidiabetic effect obtained for each experimental group, demonstrating that teuhetenone A has an activity comparable to insulin. In the diabetic mice, the effect of glibenclamide was not significant (not shown).

## 3. Discussion

Diabetic patients, even in urban areas in Mexico, use medicinal plants to control their disease, sometimes with complementation by conventional therapies. The species used can be obtained fresh from markets or in packaged form. This is the case with *T. diffusa* (damiana), which is consumed and sold throughout the country for various health conditions and complications, including diabetes. For this reason, a study of its pharmacological properties is important. In the literature, we found contradictory information about the hypoglycemic effect of *T. diffusa*. In 1984, Pérez et al. evaluated an aqueous extract in mice with alloxan-induced diabetes. In their study, they administered the extract either orally or intraperitoneally, and glucose was measured once using a glucose oxidase method five hours after the extract was administered. The *T. diffusa* extract showed good hypoglycemic activity by either method of administration [17]. Aguilar et al. reported on a hypoglycemic effect from an aqueous extract of *T. diffusa* (4 mL/kg) in rabbits that were made temporarily hyperglycemic. *T. diffusa* extract significantly decreased the hyperglycemic peak by 15.9% (*p* < 0.005) [15]. However, in 2002, the same investigators reported that there was no significant decrease in glucose levels in normoglycemic mice or mice with alloxan-induced diabetes after administration of a hydroalcoholic extract (500 mg/kg) of *T. diffusa* [16]. Besides, a methanolic extract of leaves of *Turnera ulmifolia* at 400 mg/kg significantly reduced blood glucose levels in normal rats and in rats with alloxan-induced diabetes [19].

We found the treatment of normal mice with a methanolic extract of *T. diffusa* promising, because their glucose levels were significantly reduced. The discrepancy between these results and those reported in the literature could be the result of various factors, because the experimental conditions between different trials were different. For this reason, we decided to follow-up these experiments with a bioassay-guided fractionation of the extract to identify the compound(s) responsible for the hypoglycemic activity.

**Figure 7:**
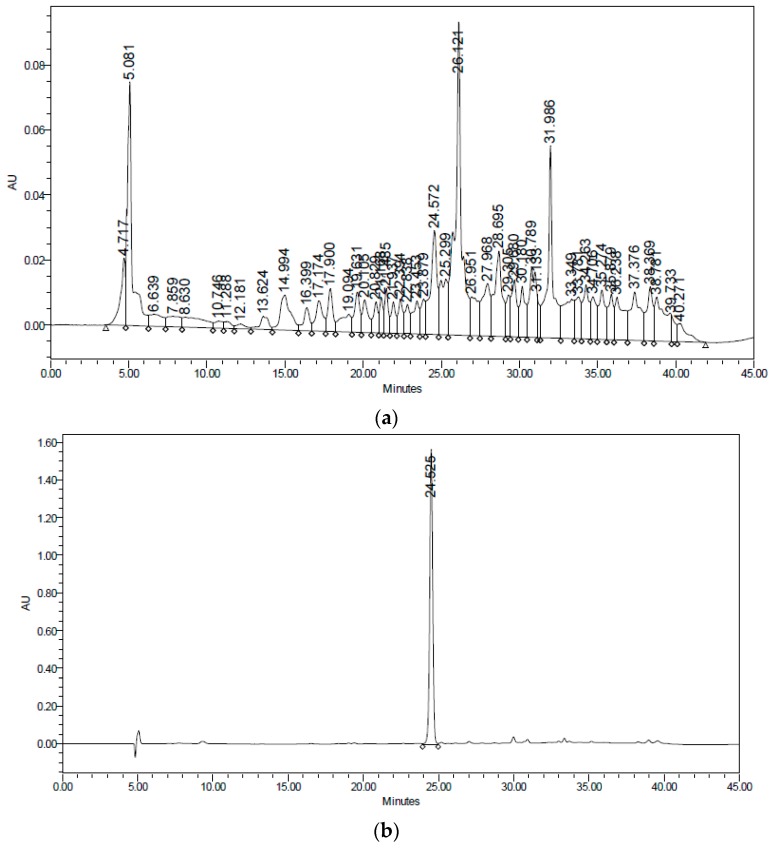
(**a**) Chromatogram of the original extract of *T. diffusa*, conditions as in text; (**b**) Chromatogram of teuhetenone A.

After the removal of chlorophyll with solid phase extraction (SPE) cartridges (Extract-Clean SPE C18-HC; 1000 mg/8 mL, Grace Davison-Alltech, Deerfield, IL), only the fraction eluted with 50% MeOH showed activity; vacuum chromatography was performed with this fraction using solvents of increasing polarity. The extract obtained with ethyl acetate showed hypoglycemic activity. Surprisingly, this fraction did not show antioxidant activity, and no strong presence of flavonoids was detected, unlike the AcOEt:MeOH fraction, in which most of the plant flavonoids were identified but were not active. The active fraction contained a major constituent, which was later identified as teuhetenone A after purification (Figure 2). A chromatographic analysis of the methanolic extract showed that teuhetenone A was present in the original extracts (retention time 24.5 min) and was not an artifact produced during the purification process (Figure 7).

The structure for teuhetenone A was assigned on the basis of spectroscopic evidence, comparison with literature values [13,20,21], and the following consideration: the ^1^H-NMR spectrum showed the presence of two uncoupled methyl groups at δ 1.43 and 1.31, and a singlet at 6.35 ppm. In DEPT experiments, we found two primary and five secondary carbon atoms, and one tertiary carbon atom in the olefin region; as well as four quaternary carbon atoms, one as a carbonyl and the other in the olefin region. HSQC and HMBC experiments assisted the elucidation, and four diastereotopic carbon atoms were revealed. Selective ^1^H-NOESY to the methyl signals allowed us to confirm that C-11 and C-12 were on the same side of the plane. Irradiation of H-11 (1.31 ppm) dipolar coupling with H-1b, H-3a, H-8b, H-9a, and H-12 was observed; moreover, irradiation of H-12 (1.43 ppm) dipolar coupling with H-3b, H-6, and H-11 was found. Optical rotation was also similar to that reported for this molecule.

Teuhetenone A is a terpenoid that has been previously isolated from *T. diffusa* and other plants, but to our knowledge, no hypoglycemic activity has been attributed to it thus far [13,20]. In the literature, it is possible to find some references to its biological activity: it has low activity in an antiaromatase test, low activity as an antifungal, it has slightly neuroprotective properties, and it moderately inhibited the production of nitric oxide by macrophages (IC_50_ = 12.93 μg/mL, positive control IC_50_ = 0.17 μg/mL) [22,23,24,25]. However, the literature is not conclusive.

Before evaluating the hypoglycemic activity of the isolated compound, the glycemic state of normoglycemic murine models was determined. Mean basal glycemic levels in the mice were in the range of 140–155 mg/dL, significantly higher than the 80–90 mg/dL after fasting for 12 h (not shown). To avoid biochemical and endocrine alterations related to fasting, or hypoglycemia induced by hypoglycemic agents and insulin that could be lethal in mice deprived of food, the mice were maintained on water and food *ad libitum* during the night before the hypoglycemic test was conducted. However, to avoid a hyperglycemic event from intake while evaluating the hypoglycemic effect, food was withdrawn, maintaining the mice on water alone during the six-hour period of the test.

As seen in Figure 3, although the hypoglycemic activity of teuhetenone A and insulin appeared similar, the observed kinetics for both compounds were very different. While insulin reached a reversible maximum hypoglycemic activity one hour after administration, the decrease in glycemia with teuhetenone A was gradual and did not reverse for at least six hours after administration, potentially maintaining an “antidiabetic” effect for longer and thereby reducing the frequency of administration required for long-term control of glycemia.

The hypoglycemic activity of teuhetenone A in the normoglycemic mice suggested possible antidiabetic activity; however, their response may have resulted from an insulin-related mechanism. Therefore, to discriminate the hypoglycemic effect of the endogenous insulin by teuhetenone A in the mice and to determine the antidiabetic effect, diabetes was induced pharmacologically with 200 mg/kg alloxan (intraperitoneally) in eight groups of five mice each. In these groups of mice, insulin secretion was limited by the pancreotoxic effect of alloxan, so glycemia increased from 140–155 to 340–380 mg/dL (Figure 5). At 1 mg/kg, teuhetenone A showed an antidiabetic effect similar to that observed with insulin, but the glucose levels did not reverse during the period evaluated (Figure 3 and Figure 5). Injection of the mice with alloxan produced a significant increase in glucose levels compared with that in a nondiabetic control group, because in these mice, insulin secretion was limited by the destruction of pancreatic β cells by alloxan [21], which is the mechanism for the hyperglycemic state of the mice. Therefore, this model is useful for evaluating antidiabetic effects. The results obtained in mice with diabetes induced by alloxan suggest that the antidiabetic action of teuhetenone A is similar to insulin, taking into account that with both substances, the glucose level decreases by approximately 35%.

Analysis of the area under the curve (AUC) regarding the kinetics of both insulin and teuhetenone A (hypoglycemic and antidiabetic) (Figure 3 and Figure 5) confirmed the effect described for teuhetenone A. The AUCs obtained for the normoglycemic control mice were 5.17 ± 0.11 for the vehicle and 4.92 ± 0.09 for insulin, which were similar to the values obtained for teuhetenone A (4.93 ± 0.08). Moreover, in diabetic mice, the AUCs for vehicle, insulin, and teuhetenone A were 5.93 ± 0.04, 5.48 ± 0.05, and 5.43 ± 0.03, respectively. These results suggest that teuhetenone A has hypoglycemic and antidiabetic effects very similar to those shown by insulin. Therefore, the hypoglycemic activity shown by teuhetenone A is interesting because it is more potent than that of glibenclamide (Figure 4) and as active as insulin (Figure 5).

Neither the extracts nor the isolated teuhetenone A significantly inhibited the activity of α-glucosidase in vitro, which is consistent with the literature, so the mechanism of action of this terpenoid is probably different from that of hypoglycemic agents that inhibit the absorption of carbohydrates from the diet.

The isolated teuhetenone A was not toxic to Vero cells, consistent with previous reports of inactivity on HepG2 and Hep3B liver, MCF-7 and MDA-MB-231 breast, and A-549 lung carcinoma cell lines [26].

We conclude that teuhetenone A, or a standardized extract containing it, would be a good candidate for an herbal-based antidiabetic therapy.

## 4. Materials and Methods

### 4.1. Biological Materials and Reagents

*T. diffusa* (leaves and stems) were collected in Montemorelos, Nuevo León, Mexico in November 2014 (latitude 25.1186, longitude −99.7865). A voucher specimen (No. 23569) was identified and deposited in the herbarium at the Faculty of Biology, Universidad Autónoma de Nuevo León, Mexico.

Vero cells were used to assess cytotoxicity with Dulbecco’s modified Eagle’s medium (DMEM, Advanced) supplemented with 10% fetal bovine serum, 1% antibiotics (penicillin G/streptomycin 10000 units/mL), and 1% l-glutamine 200mM. All the solvents used for extraction and purification were analytical grade and were purchased from Fermont (Monterrey, Nuevo León, Mexico). HPLC-grade methanol (MeOH) was purchased from JT Baker (Fisher Scientific, Fair Lawn, New Jersey). Extract-Clean SPE C18-HC 1000 mg/8 mL cartridges were purchased from Grace Davison-Alltech (Deerfield, Illinois). TLC was conducted on plates precoated with silica gel 60 F_254_ at a thickness of 0.2 mm with an aluminum support (Merck, Darmstadt, Germany). MilliQ deionized water was obtained with an Elga II MilliQ system (Veolia, Mexico). Mobile phases were filtered through a 0.45 μm nylon filter before use (Waters Corp., Milford, MA). The samples were filtered through nylon Acrodiscs (Waters Corp, Milford, MA, USA).

### 4.2. Isolation and Purification

Before extraction, the aerial parts of *T. diffusa* were dried in the shade at room temperature and then crushed into a fine powder. Then, 500 g of the dried selected plant was crushed into a powder and extracted with MeOH at room temperature (3 × 800 mL × 1 h × 200 rpm) with a Heidolph Unimax 1010 shaker. All the extracts were pooled together and evaporated to dryness *in vacuo* to yield a viscous mass, which weighed 60 g. The chlorophyll contents were eliminated using SPE C18 cartridges, and the samples were eluted with 8 mL of 50%, 70%, and 100% MeOH. Five grams of the fraction obtained using 50% MeOH was subjected to silica vacuum liquid chromatography (silica gel 60 G for thin layer chromatography Merck Millipore, 5 g for sample adsorption, 50 g for the column, Hirsch funnel with pore size M using Cl_2_CH_2_, AcOEt, AcOEt:MeOH (1:1), and MeOH as eluents (400 mL of each solvent). The fraction eluted with AcOEt (158 mg) was active and was further separated by gravitational chromatography), eluted successively with hexane-AcOEt (3:1, 70 mL) and hexane-AcOEt (2:1, 150 mL). We collected 20 fractions, each containing 10 mL. Fractions 17 and 18 were pooled and evaporated to dryness in vacuo to yield 3.7 mg of yellow oil. The purity of the oil was assessed by TLC, HPLC-DAD, and ^1^H-NMR. The proposed structure was established based on the NMR results and comparisons with published results.

### 4.3 Instrumental Analysis

Chromatograms were obtained using a Waters 600 Series HPLC system including a 717 autosampler, 2996 diode array detector, and binary pump 1525. HPLC conditions were as follows: Hypersil Gold C-18 column (5 μm, 150 × 4.6 mm; Thermo Scientific Waltham, MA, USA); solvent system: A = 0.1% aqueous HCOOH solution, B = MeOH, eluted with a gradient from 70% to 40% A over 20 min, held at 40% A for 5 min, after which the proportion of A was changed to 30% over 15 min before being returned to the initial conditions for 5 min; flow 0.4 mL/min; injection volume 10 μL; and concentration of the sample 1 mg/mL in MeOH. Optical rotation was determined using a Perkin-Elmer 341 polarimeter. To determine NMR spectra, a Bruker Avance DPX400 model 400 MHz NMR spectrometer was used. The mass of teuhetenone A was obtained in a UHPLC Ultimate 3000 (Dionex, Thermo Scientific) system including an auto sampler, quaternary pump, and variable wavelength detector interfaced with an LCQFleet (Thermo Scientific) mass spectrometer equipped with an ESI ionization chamber. Separation was performed on a Hypersil Gold C-18 column, 50 × 2.1 mm, 1.9 µm (Thermo Scientific) at 25 °C using 1% aqueous HCOOH (A) in MeOH (B) as the mobile phase. The gradient profile used was as follows: 30% to 45% of B over 1.8 min, changed to 60% B over 4.6 min, and finally to 100% B over 2.2 min; flow 0.2 mL/min; injection volume 0.7 µL; and concentration of the sample 1mg/mL in MeOH. The first detections were done at 280 and 350 nm, followed by a second detection using mass spectrometry. Mass analyses were obtained in the negative ion mode. The mass spectrometer was programmed to perform three consecutive scans: full mass (*m/z* 200–2000), MS2 of the most abundant ion in the full mass, and MS3 of the most abundant ion in the MS2. The source voltage was 5 kV, and the capillary voltage and temperature were 14 V and 275 °C, respectively. Nitrogen was used as the sheath and auxiliary gas at 30 and 20 units, respectively. The normalized collision energy using helium as collision gas was 35%.

### 4.4. Alpha-Glucosidase Assay

The α-glucosidase assay was performed as described by Apostolidis et al. with some modifications [27]. One milligram of the obtained fraction was dissolved in 500 μL of phosphate buffer (100 mM, pH 6.8). Various concentrations of the solution (final concentrations between 20.6 to 330 μg/mL in 33 μL) were mixed with 17 μg of p-NPG (to give a final concentration 33.6 μg/mL) and incubated for 5 min at 37 °C. Subsequently, 17 μL of α-glucosidase was added (to give a final concentration 46.75 mU/mL) and incubated for 17.5 min at 37 °C. Finally, 133 μL of sodium carbonate (final concentration 66.5 μm) was added. Absorbance was measured on a plate-reading spectrophotometer at 405 nm. In each experiment, a 100% control of enzymatic activity was included, in which 33 μL of phosphate buffer 100 mM, pH 6.8 was included, instead of sample or standard. Also included was a sample blank or standard, containing only 17 μL of buffer but no enzyme. The absorbance of the sample blank or standard was subtracted from the absorbance values of its sample or standard. All experiments were performed in triplicate. The percentage of enzyme inhibition was calculated using the following formula.

(1) $$\%\;enzyme\;inhibition=100-\left[\frac{\left(Abs\;sample-Abs\;blank\right)}{Abs\;control\;100\%}\;\times 100\right]$$

The percentage reduction was plotted according to sample concentrations. From the curve, the concentration that inhibited 50% of the activity (IC_50_) was interpolated.

### 4.5. Cytotoxic Activity

Cytotoxicity evaluation was performed using a 3-(4,5-dimethylthiazol-2-yl)-2,3-diphenyltetrazolium (MTT) bromide salt reduction test as described by Mosmann et al. in 1983 [28] with some modifications. To perform the assay, 44,000 Vero cells per well were added to 96-well plates; after 24 h, the medium was removed, and 100 μL of teuhetenone A dissolved in culture medium at concentrations of from 3.9 to 500 μg/mL was added. The plates were incubated at 37 °C for 48 h under an atmosphere of 5% CO_2_, and the cells were then washed twice with phosphate-buffered saline. After washing, 200 μL MTT 0.5 mg/mL was added and incubated with the cells for an additional three hours. After this time, the supernatant was decanted, and 200 μL of DMSO was added; the mixture was shaken for eight minutes at 25 rpm and read spectrophotometrically at 570 nm in a plate reader.

### 4.6. In Vivo Experiments

To assess hypoglycemic activity, both normoglycemic and diabetic CD1 mice (24 to 40 g) were used. Male CD1 mice were maintained for seven days in an acclimation period before the experiment. Mice were maintained in the animal experimental area under standard conditions (12 h light/dark cycle, at 23 ± 2° C, 65% humidity) through the acclimation and during the experiment. Animals were weighed and allocated in groups of five animals in polycarbonate cages of 44 × 28 × 15 cm according with the treatment. Pelleted food and water were offered *ad libitum*. All experimental protocols were approved by the Institutional Animal Care and Use Committee of the Faculty of Medicine (UAEM) and comply with the applicable Mexican Official Norm (NOM-062-ZOO-1999), “Technical Specifications for the Care and Use of Laboratory Animals”, as well as all applicable federal and institutional regulations. Diabetes was induced by administering 200 mg/kg alloxan. A blood sample to measure glycemia was obtained by cutting 1 mm from the tip of the tail. Glucose concentration was determined by the use of a commercial glucometer (Optium Xceed, Abbott) and expressed as mg/dL. Mice with glycemia equal to or greater than 250 mg/dL were considered diabetic. Five groups of five mice each were formed for both normoglycemic and diabetic mice: vehicle control (water), insulin positive control 5 IU/kg, a positive control of glibenclamide 5 mg/kg, and the isolated teuhetenone A (1 and 5 mg/kg) were administered. Basal glycemia was measured before administration of the tested substance or vehicle (control group). The test substances diluted to 100 μL were administered intraperitoneally, and blood glucose readings were made every hour for six hours. Once the evaluation was over, the obtained experimental data were normalized with respect to the basal glycemia of each mouse, and the means and standard errors were plotted and adjusted to a sigmoid equation.

### 4.7. Statistical Analysis

All the results were processed using measures of central tendency (mean) and dispersion (standard error). Statistical differences between results from each experimental group were evaluated with a Student *t* test or one-way ANOVA using GraphPad Prism software, version 6.05.

## Figures and Tables

**Table 1:** Inhibitory effect of teuhetenone A on α-glucosidase (IC_50_, μg/mL) ^a,b^.

Compound	IC_50_
Teuhetenone A	>330
Acarbose	210.5 ± 7.30

^a^ Average of the triplicate (± SD). ^b^ Acarbose as positive control.
